# Cefiderocol treatment for patients infected by *Stenotrophomonas maltophilia*, *Burkholderia cepacia* complex and *Achromobacter* spp.: subgroup analysis from the PERSEUS study

**DOI:** 10.1007/s10096-025-05109-5

**Published:** 2025-03-24

**Authors:** Julian Torre-Cisneros, Ricard Ferrer, Carmen De La Fuente Martos, Jessica Sarda, A. Javier Gonzalez Calvo, Stefano Verardi, Andreas Karas, Alex Soriano

**Affiliations:** 1 Maimonides Institute for Biomedical Research, Córdoba, Spain; 2 Centro de Investigación Biomédica en Red de Enfermedades Infecciosas, Instituto de Salud Carlos III, Madrid, Spain; 3 Infectious Diseases Service, Hospital Universitario Reina Sofía, Córdoba, Spain; 4 Department of Medical and Surgical Sciences, University of Córdoba, Córdoba, Spain; 5 Intensive Care Department, Hospital Universitari Vall d’Hebrón, SODIR Research Group, Vall d’Hebron Institut de Recerca, Universitat Autònoma de Barcelona, Barcelona, Spain; 6 Critical Care Service, Hospital Universitario Reina Sofía, Córdoba, Spain; 7 Shionogi S.L.U, Calle de Serrano 45, Madrid, 28001 Spain; 8 Shionogi BV, London, UK; 9 Department of Infectious Diseases, University of Barcelona, Hospital Clinic of Barcelona, Barcelona, Spain; 10 IDIBAPS, Institut d’Investigacions Biomèdiques Agustí-Pi Sunyer, Barcelona, Spain

**Keywords:** Cefiderocol, *Stenotrophomonas maltophilia*, *Burkholderia cepacia* complex, *Achromobacter* spp, Non-fermenting Gram-negative bacteria

## Abstract

**Purpose:**

This subgroup analysis of the PERSEUS study aimed to describe the effectiveness of cefiderocol treatment in the early access programme in Spain in patients infected by *Stenotrophomonas maltophilia*, *Burkholderia cepacia* complex (Bcc) or *Achromobacter* species.

**Methods:**

In the retrospective, observational, multicentre PERSEUS study in Spain, the effectiveness and safety of cefiderocol treatment administered for at least 72 h up to 28 days in patients infected by Gram-negative bacteria, except *Acinetobacter* spp., in the early access programme was investigated. Patient demographics and baseline clinical characteristics, cefiderocol use, clinical cure at end of treatment, all-cause mortality at Day 28 were the main outcomes.

**Results:**

A total of 20 patients had *S. maltophilia* infections, and 14 patients had other rare glucose non-fermenters (Bcc 8, *Achromobacter* spp. 5, *Ralstonia mannitolilytica* 1). The median (interquartile range [IQR]) age was 60.5 (48.0–65.5) years and 49.5 (33.0–59.0) years for patients with *S. maltophilia* and other rare non-fermenters, respectively. The majority of patients had respiratory tract infections (*S. maltophilia* 55%; other rare non-fermenters 71.4%), and median (IQR) duration of cefiderocol treatment was 10.0 (6.5–13.5) days and 8.0 (6–14) days, respectively. Clinical cure rates were 70%, 62.5% and 80.0% for patients with *S. maltophilia*, Bcc and *Achromobacter* spp., respectively. Corresponding 28-day all-cause mortality rates were 30.0%, 37.5% and 40.0%, respectively. One patient with *R. mannitolilytica* had clinical cure and survived to Day 28.

**Conclusions:**

Cefiderocol is an important addition to the limited treatment options for patients infected by these rare glucose non-fermenting Gram-negative bacteria.

**Trial registration:**

ClinicalTrials.gov: NCT05789199 (Registration date: 16 February 2023).

**Supplementary Information:**

The online version contains supplementary material available at 10.1007/s10096-025-05109-5.

## Introduction

*Stenotrophomonas maltophilia*, *Burkholderia cepacia* complex (Bcc) and *Achromobacter* spp. are rare, opportunistic, glucose non-fermenting Gram-negative pathogens that can colonise the mucosa of in-patients who, typically, are under high antibiotic pressure, resulting in the potential for severe infections linked to increased risk of mortality and morbidity in vulnerable patients [1–12]. These species are frequently resistant to beta-lactam antibiotics, including penicillins, cephalosporins, carbapenems and beta-lactam–beta-lactamase inhibitor combinations, as well as agents in other antibiotic classes [3–5, 7, 13–16]. Thus, treatment of infections caused by *S. maltophilia*, Bcc or *Achromobacter* spp. is challenging [5, 7, 17–19].

Cefiderocol, a siderophore cephalosporin, has demonstrated excellent in vitro activity against *S. maltophilia*, Bcc and *Achromobacter* spp. isolates with low MIC values in multinational surveillance studies [14, 20–23]. The observational PERSEUS study investigated the effectiveness of cefiderocol treatment in the cefiderocol early access programme (EAP) in Spain and enrolled patients with infections caused by Gram-negative bacteria, excluding *Acinetobacter* spp [24]. In the PERSEUS study, clinical cure was achieved in 80.5% of patients and all-cause mortality at Day 28 was 21.5% overall [24].

In this subgroup analysis, we describe the clinical and microbiological characteristics of *S. maltophilia* and other rare, non-fermenting Gram-negative bacterial infections, including resistance and treatment patterns, in patients enrolled in the PERSEUS study, and report on the clinical outcomes and use of cefiderocol in this subset of patients.

## Methods

### Ethics and regulations

In Spain, access to locally unapproved medications can be granted in special circumstances following a Royal Decree (1015/2009), with each case considered on its own merits for approval by the Spanish Agency of Medicines. Prior to study start, the PERSEUS study was approved centrally by the Institutional Review Board of Hospital La Princesa, Madrid, under Royal Decree 957/2020 (3 November 2020). The study was conducted according to all legal and regulatory requirements, the International Conference on Harmonisation Good Clinical Practice E6 guidelines and the Declaration of Helsinki. Medical record data were collected and anonymised to protect patients’ personal information. In accordance with Spanish regulations, patient consent was waived due to the retrospective design of the study as patients completed their treatment prior to study initiation; therefore, the study represented no harm for the participants.

The design of the PERSEUS study, eligibility criteria, populations, outcomes, variables, and definitions, and statistical analysis have been described in detail by Torre-Cisneros J, et al. [24].

## Results

### Patient disposition

Of the 261 patients included in the primary analysis population of the PERSEUS study, 34 were included in this subgroup analysis, of whom 20 patients had *S. maltophilia* infections and 14 had other glucose non-fermenting Gram-negative spp. infections, including 8 with Bcc infections, 5 with *Achromobacter* spp. infections and 1 with *Ralstonia mannitolilytica* infection.

### Patient demographics and baseline characteristics

Across the different pathogen groups, most patients were male (except for those with *Achromobacter* spp. infections). Patients were generally young, particularly patients with Bcc, who had a median age of 41.0 years. The median CCI ranged between 1.0 and 3.0, and around three-quarters of patients had an underlying illness (Table 1, Table S1). Cancer and moderate or severe renal disease were more common in patients with *S. maltophilia* than in patients with infections caused by the other non-fermenting groups (Table S1). Structural lung disease was the most common condition in patients with other non-fermenting Gram-negative bacterial infections (i.e. those with Bcc infections). Other comorbidities included diabetes mellitus, cerebrovascular disease, and peripheral vascular disease (Table S1).

**Table 1:** Patients’ baseline demographics and clinical characteristics by non-fermenting Gram-negative bacterial species

	*S. maltophilia*	Other non-fermenting Gram-negative bacteria		
	*N* = 20	Total *N* = 14^a^	Bcc *N* = 8	*Achromobacter* spp. *N* = 5
Age (years), median (IQR)	60.5 (48.0–65.5)	49.5 (33.0–59.0)	41.0 (28.5–54.0)	59.0 (56.0–61.0)
Sex (male), *n* (%)	14 (70.0)	9 (64.3)	7 (87.5)	2 (40.0)
CCI, median (IQR)	3.0 (2.0–5.0)	2.0 (1.0–3.0)	1.0 (1.0–3.0)	3.0 (2.0–3.0)
SOFA, median (IQR)	9.0 (8.0–11.0)	6.0 (4.0–11.0)	5.5 (3.0–11.0)	5.0 (4.0–10.0)
APACHE II, median (IQR)	15 (6.0–24.0)	15 (9.0–22.0)	14.0 (9.0–19.0)	16.0 (8.0–34.0)
ICU, *n* (%)	15 (75.0)	11 (78.6)	7 (87.5)	3 (60.0)
Mechanical ventilation at baseline, *n* (%)	11 (55.0)	10 (71.4)	6 (75.0)	3 (60.0)
Symptomatic COVID-19 during hospitalisation, *n* (%)	4 (20.0)	1 (7.1)	0 (0)	1 (20.0)
Ventilation for COVID-19-related symptoms, *n/N* (%)	4/4 (100)	1/1 (100)	0 (0)	1/1 (100)
Septic shock, *n* (%)	6 (30.0)	3 (21.4)	1 (12.5)	1 (20.0)
ECMO, *n* (%)	1 (5.0)	2 (14.3)	1 (12.5)	1 (20.0)
RRT, *n* (%)	5 (25.0)	6 (42.9)	3 (37.5)	2 (40.0)
Creatinine clearance < 60 mL/min, *n/N* (%)^b^	3/15 (20.0)	4/8 (50.0)	4/5 (80.0)	0/3 (0)
Immunosuppressed, *n* (%)^c^	11 (55.0)	9 (64.3)	5 (62.5)	3 (60.0)
Transplant recipient, *n* (%)	9 (45.0)	8 (57.1)	5 (62.5)	2 (40.0)
Solid	3/9 (33.3)	7/8 (87.5)	5/5 (100)	1/2 (50.0)
Haematopoietic	6/9 (66.7)	1/8 (12.5)	0/5 (0)	1/2 (50.0)
Primary infection site, *n* (%)				
Respiratory	11 (55.0)	10 (71.4)	7 (87.5)	2 (40.0)
Bloodstream (catheter related)	4 (20.0)	1 (7.1)	0 (0)	1 (20.0)
Bloodstream (unknown source)	1 (5.0)	0 (0)	0 (0)	0 (0)
Urinary	1 (5.0)	2 (14.3)	0 (0)	2 (40.0)
Intra-abdominal	2 (10.0)	1 (7.1)	1 (12.5)	0 (0)
Skin and soft tissue	0 (0)	0 (0)	0 (0)	0 (0)
Bone and joint	0 (0)	0 (0)	0 (0)	0 (0)
Other	1 (5.0)^d^	0 (0)	0 (0)	0 (0)
Secondary bacteraemia, *n* (%)	4 (20.0)	3 (21.4)	0 (0)	2 (40.0)
Polymicrobial infection, *n* (%)^e^	3 (15.0)	1 (7.1)	1 (12.5)	0 (0)
Previous colonisation, *n* (%)	9/19^f^ (47.4)	7/13^f^ (53.8)	5 (62.5)	2 (40.0)

Bcc, *Burkholderia cepacia* complex; CCI, Charlson Comorbidity Index; COVID-19, coronavirus disease-2019; ECMO, extracorporeal membrane oxygenation; NF-GN, non-fermenting Gram-negative; RRT, renal replacement therapy

^a^One patient had *Ralstonia mannitolilytica* infection (further details not shown), 8 patients had Bcc, 5 patients had *Achromobacter* spp

^b^Excludes RRT; denominator excludes the missing data

^c^Transplant recipient, immunosuppressive treatment (e.g. high-dose corticosteroids, calcineurin inhibitors, anti-CD20, IL-1 inhibitors and IL-6 inhibitors)

^d^Includes mediastinitis (*n* = 1)

^e^Primary pathogen in polymicrobial infections, for which cefiderocol was requested, was confirmed by the treating physician

^f^Information on previous colonisation was available for 19 *S. maltophilia* isolates and 13 other NF-GN isolates

Between 60.0% and 87.5% of patients required admission to an ICU (Table 1). Across the pathogen groups, the median durations of hospital and ICU stays were 75.0–97.5 days and 25.0–89.0 days, respectively (Table S2). Over half of patients in each pathogen group (≥ 55%) were mechanically ventilated at baseline and/or were immunosuppressed. Additionally, 50% of patients (17/34) were solid organ or haematopoietic transplant recipients. Septic shock was reported in 12.5–30.0% of patients, and 25–42.9% of patients were receiving renal replacement therapy (Table 1). The median SOFA score was 9.0 in patients with *S. maltophilia* and 6.0 for other rare non-fermenters (Table 1).

In most patients, the primary infection site was respiratory (40.0–87.5% of patients across all pathogen groups) and infections were mainly monomicrobial (Table 1). Unlike patients with other pathogens, those with Bcc infections did not have secondary bacteraemia. There was a high rate of previous colonisation with the same pathogen (Table 1).

The reported susceptibility data showed that most isolates across these species were resistant to standard antibiotic treatments. Among *S. maltophilia* and other non-fermenting Gram-negative pathogens, susceptibility rates (combined susceptible + intermediate [susceptible, increased exposure by EUCAST]) to trimethoprim-sulfamethoxazole were 62.5% and 25.0%, respectively, 50.0% and 25.0%, respectively, to colistin, and 44.4% of *S. maltophilia* were susceptible to levofloxacin. Minocycline susceptibility rates of 80.0% and 66.7% were reported for *S. maltophilia* and other non-fermenting Gram-negative pathogens, respectively, while respective rates of susceptibility to ceftazidime-avibactam and ceftazidime were 0% and 14.2% for *S. maltophilia* and 20.0% and 0% for other non-fermenting Gram-negative pathogens (Table S3). All isolates tested were resistant to meropenem, ciprofloxacin and ceftolozane-tazobactam (Table S3).

### Treatment patterns

Over 60% of patients in each pathogen group had received prior antibiotics (Table 2). The median number of courses and median duration of prior antibiotics were 2.0–2.5 and 6.3–12.5 days, respectively (Table 2). The most common (50.0%) prior antibiotic used for patients with *S. maltophilia* was trimethoprim-sulfamethoxazole. For patients with Bcc, meropenem and colistin, and for patients with *Achromobacter* spp., meropenem, ceftazidime-avibactam, and colistin were administered most commonly (Table S4).

**Table 2:** Pattern of cefiderocol use, prior and concomitant antibiotic use by Gram-negative bacterial species

	*S. maltophilia*	Other non-fermenting Gram-negative bacteria		
	*N* = 20	Total *N* = 14^a^	Bcc *N* = 8	*Achromobacter* spp. *N* = 5
Prior antibiotics, *n* (%)^b^	16 (80.0)	10 (71.4)	7 (87.5)	3 (60.0)
Number of prior courses of antibiotic treatments, median (IQR)	2.5 (2.0–3.0)	2.0 (1.0–3.0)	2.0 (1.0–4.0)	2.0 (1.0–3.0)
1, *n* (%)	1/16 (6.3)	3/10 (30.0)	2/7 (28.6)	1/3 (33.3)
2, *n* (%)	7/16 (43.7)	3/10 (30.0)	2/7 (28.6)	1/3 (33.3)
≥ 3, *n* (%)	8/16 (50.0)	4/10 (40.0)	3/7 (42.9)	1/3 (33.3)
None, *n* (%)	3 (15.0)	3 (21.4)	1 (12.5)	1 (20.0)
Unknown, *n*	1	1	0	1
Duration of prior antibiotic treatment (days), median (IQR)	9.3 (5.6–15.9)	11.8 (6.0–23.0)	12.5 (6.0–23.0)	6.3 (3.0–49.5)
≤ 3, *n* (%)	1/16 (6.3)	2/10 (20.0)	1/7 (14.3)	1/3 (33.3)
4–7, *n* (%)	5/16 (31.3)	2/10 (20.0)	1/7 (14.3)	1/3 (33.3)
> 7, *n* (%)	10/16 (62.3)	6/10 (60.0)	5/7 (71.4)	1/3 (33.3)
Rationale for administration of cefiderocol, *n* (%)^c^				
Resistance to all tested antibiotics	10 (50.0)	11 (78.6)	6 (75.0)	4 (50.0)
Treatment failure of prior antibiotics	12 (60.0)	5 (35.7)	5 (62.5)	0 (0)
Adverse events to other susceptible antibiotics	4 (20.0)	0 (0)	0 (0)	0 (0)
Other	5 (25.0)	1 (7.1)	0 (0)	1 (20.0)
Cefiderocol as first-line therapy, *n* (%)	3 (15.0)	3 (21.4)	1 (12.5)	1 (20.0)
Duration of cefiderocol treatment (days), median (IQR)	10.0 (6.5–13.5)	8.0 (6.0–14.0)	9.0 (6.5–14.0)	8.0 (4.0–8.0)
Combination therapy given with cefiderocol, *n* (%)^d^	9 (45.0)	9 (64.3)	7 (87.5)	2 (40.0)
Number of antibiotics concomitantly with cefiderocol, *n* (%)				
1	5/9 (55.6)	4/9 (44.4)	3/7 (42.9)	1/2 (50.0)
2	1/9 (11.1)	2/9 (22.2)	1/7 (14.3)	1/2 (50.0)
≥ 3	3/9 (33.3)	3/9 (33.3)	3/7 (42.9)	0 (0)
Cefiderocol dosing, *n* (%)				
Every 4 h	0 (0)	0 (0)	0 (0)	0 (0)
Every 6 h	2 (10.0)	1 (7.1)	1 (12.5)	0 (0)
Every 8 h	15 (75.0)	13 (92.9)	7 (87.5)	5 (100)
Every 12 h	3 (15.0)	0 (0)	0 (0.0)	0 (0)
Other	0 (0)	0 (0)	0 (0)	0 (0)

Bcc, *Burkholderia cepacia* complex; GN, Gram-negative; NF-GN, non-fermenting Gram-negative

^a^One patient had *Ralstonia mannitolilytica* infection (further details not shown), 8 patients had Bcc, 5 patients had *Achromobacter* spp

^b^Data are shown for patients with a full data set

^c^Not mutually exclusive; physicians could select ≥ 1 option

^d^Includes antibiotics with Gram-negative coverage that have been started before, concomitantly or during the same treatment period

Cefiderocol was infrequently administered as first-line therapy, and it was used mainly following reported resistance and treatment failure with all other tested antibiotics (Table 2). Cefiderocol combination treatment was given to between 40.0% and 87.5% of patients (the latter for Bcc) (Table 2). Concomitant antibiotics included mainly trimethoprim-sulfamethoxazole, colistin, tigecycline and ceftazidime-avibactam (Table S5). The median duration of cefiderocol treatment across pathogen types was 8.0–10.0 days (Table 2).

### Outcomes

The clinical cure rate was 70.0% for *S. maltophilia* infections and 71.4% for infections caused by other non-fermenting Gram-negative bacteria, including rates of 62.5% for patients with Bcc and 80.0% for patients with *Achromobacter* spp. infections (Table 3). For 11 patients specifically with respiratory tract infections caused by *S. maltophilia*, the clinical cure rate was 63.6% (7/11). The mortality rate for patients with infections caused by *S. maltophilia* was 30.0% (Table 3), and 36.4% (4/11) in the 11 patients with *S. maltophilia* respiratory tract infections.

**Table 3:** Clinical cure at end of treatment, all-cause mortality and clinical success rates by Gram-negative bacterial species

	Overall	*S. maltophilia*	Other non-fermenting Gram-negative bacteria		
	*N* = 261^a^	*N* = 20	Total *N* = 14^b^	Bcc *N* = 8	*Achromobacter* spp. *N* = 5
Clinical cure, *n* (%)	210 (80.5)	14 (70.0)	10 (71.4)	5 (62.5)	4 (80.0)
Patients with prior trimethoprim-sulfamethoxazole treatment	N/A	7/10 (70.0)	N/A	N/A	N/A
Patients without prior trimethoprim-sulfamethoxazole treatment	N/A	7/10 (70.0)	N/A	N/A	N/A
28-day mortality, *n* (%)	56 (21.5)	6 (30.0)	5 (35.7)	3 (37.5)	2 (40.0)
Patients with prior trimethoprim-sulfamethoxazole treatment	N/A	3/10 (30.0)	N/A	N/A	N/A
Patients without prior trimethoprim-sulfamethoxazole treatment	N/A	3/10 (30.0)	N/A	N/A	N/A
Clinical success, *n* (%)	220 (84.3)	14 (70.0)	10 (71.4)	5 (62.5)	4 (80.0)
Patients with prior trimethoprim-sulfamethoxazole treatment	N/A	7/10 (70.0)	N/A	N/A	N/A
Patients without prior trimethoprim-sulfamethoxazole treatment	N/A	7/10 (70.0)	N/A	N/A	N/A

Bcc, *Burkholderia cepacia* complex; N/A, not applicable; NF-GN, non-fermenting Gram-negative

^a^Published previously in reference 24

^**b**^One patient had *Ralstonia mannitolilytica* infection, and achieved clinical cure, clinical success and survived to Day 28; 8 patients had Bcc, 5 patients had *Achromobacter* spp

Among patients with *S. maltophilia* infections, prior trimethoprim-sulfamethoxazole treatment did not influence rates of clinical cure and mortality (Table 3).

One patient with *R. mannitolilytica* infection achieved clinical cure and survived to Day 28.

## Discussion

The current data provide valuable information on the effectiveness of cefiderocol treatment for infections caused by rare non-fermenting Gram-negative species in patients with immunosuppression, pulmonary conditions, or malignancies.

In this analysis, the overall clinical cure rate ranged between 62.5% and 80% and the mortality rate between 30% and 40% among the small number of patients with the various species. By comparison, in the overall population of the PERSEUS study, cefiderocol treatment administered for a median of 10 days was highly effective, with a clinical cure rate of 80.5% and overall mortality rate of 21.5% [24]. It is worth noting that this subset of patients had clinical characteristics suggestive of more serious illness relative to the overall study population, which was confirmed by the very long hospitalisation periods and high rates of ICU admission and mechanical ventilation.

As expected in the early access programme, cefiderocol was initiated either because of resistance or treatment failure with other available antibiotics [24]. Cefiderocol treatment duration in these patients with rare non-fermenting Gram-negative infections was similar to that reported in the overall population and in patients with *Pseudomonas* spp. infections in the PERSEUS study, and followed lengthy prior treatment (median 6.3–12.5 days), with the majority of patients receiving prior antibiotics for > 7 days. The relatively greater frequency of cefiderocol use in combination with other agents in this subgroup of patients, compared with the overall population, was expected given the challenging nature of these non-fermenting bacteria (particularly Bcc) and it is highlighted in the current treatment guidelines for *S. maltophilia* [5, 7, 17–19].

There are limited treatment options for *S. maltophilia* as it is intrinsically resistant to carbapenems and other beta-lactam antibiotics [2, 3]. The relatively low in vitro susceptibility (≤ 62.5%) to trimethoprim-sulfamethoxazole among tested *S. maltophilia* isolates in the present study, compared with previous findings from Spain [25], may have been due in part to the highly select nature of the patients, having prolonged hospitalisation and failure of several previous antibiotic regimens. Data from multinational surveillance studies showed that a significant proportion of Bcc isolates were resistant to ciprofloxacin, meropenem and tigecycline, while susceptibility rates ranged between 75.6% for minocycline and 98.5% for meropenem-vaborbactam [14]. Across 267 *Achromobacter* spp. isolates from patients with cystic fibrosis, of which nearly 50% were resistant to meropenem, antibiotic susceptibility rates ranged between 0% and 70% by EUCAST breakpoints [18] and only ceftazidime-avibactam (62%) and piperacillin-tazobactam (70%) showed susceptibility rates > 50% [13].

There is limited data on the clinical efficacy of other antibiotics, with evidence largely confined to retrospective analyses and case reports. In these studies, in-hospital or 30-day mortality rates were reported as 5–56% with minocycline, 7–15% with fluoroquinolones, and 15–87% with trimethoprim-sulfamethoxazole [26–30]. In comparison with these reports, 28-day mortality rates were found to be within this range in patients in this subgroup analysis.

In the current cohort, most isolates with available reported susceptibility information were resistant to meropenem and other beta-lactam antibiotics, although a few *S. maltophilia* isolates were reported to be susceptible to minocycline, trimethoprim-sulfamethoxazole, and levofloxacin; these proportions were similar to those observed in global studies [14, 15]. It was demonstrated previously that cefiderocol had potent in vitro activity against Bcc and *Achromobacter* spp., including carbapenem-resistant strains, with the lowest MIC_90_ values across a range of antibiotic agents tested [14, 22]. Previous case reports and case series with cefiderocol involving nosocomial pneumonia, haemorrhagic pneumonia and/or bloodstream infections, and peri-prosthetic joint infections caused by *S. maltophilia*, including multidrug-resistant strains, included paediatric and adult patients with haematological malignancies, neutropenia and end-stage renal disease in whom prior antibiotic therapy had often failed [31–36]. Switching to cefiderocol monotherapy or adding cefiderocol in combination with trimethoprim-sulfamethoxazole and/or levofloxacin led to clinical improvement and microbiological eradication in most patients [31–36]. Cefiderocol-based treatment has demonstrated clinical activity against infections caused by Bcc or *Achromobacter xylosoxidans* in patients with CF and haematological malignancies, including recurrent pulmonary exacerbations, persistent bacteraemia and endocarditis, with reassuring clinical outcomes, including improved pulmonary function and high survival rates at 6 months [37–41].

In the above-mentioned real-world cases, patients infected with *S. maltophilia*, Bcc and *Achromobacter* spp. had similar clinical characteristics, risk factors, infection diagnoses and prior treatment failure to those described in the current cohort, supporting the effectiveness and activity of cefiderocol against these rare non-fermenters and the relatively high clinical cure rates in the PERSEUS study.

Based on global susceptibility results, in vivo preclinical findings and PK/PD standards, cefiderocol is currently recommended in the IDSA treatment guidance for the treatment of patients with infections caused by *S. maltophilia* in combination with other antibiotic agents to which this pathogen is susceptible [18]. Currently, there are no standard-of-care recommendations for infections caused by *Burkholderia* spp. or *Achromobacter* spp., and treatments are limited to those antibiotics that show in vitro activity in hospital laboratory testing.

## Conclusions

This small cohort of patients treated with cefiderocol comprised a complicated, critically ill population who were frequently chronically colonised by the same pathogen, and had underlying complex conditions, often linked with immunosuppressed status. In this scenario, our results support the current guidelines suggesting that cefiderocol is an appropriate alternative agent for infections caused by *S. maltophilia*, Bcc and *Achromobacter* spp., particularly when options are limited.

## Electronic supplementary material

Below is the link to the electronic supplementary material.

Supplementary Material 1

## Data Availability

Data analysed in the current analysis are not publicly available. At reasonable requests, data can be shared with investigators and researchers according to Shionogi’s clinical trial data sharing policy. This policy can be found at: https://www.shionogi.com/global/en/company/policies/shionogi-group-clinical-trial-data-transparency-policy.html.
