# Supplementary material for: Cefiderocol treatment for patients infected by Stenotrophomonas maltophilia, Burkholderia cepacia complex and Achromobacter spp.: subgroup analysis from the PERSEUS study
Source: Eur J Clin Microbiol Infect Dis. 2025 Mar 24;44(6):1367–74. doi: 10.1007/s10096-025-05109-5 (PMC12116654; doi:10.1007/s10096-025-05109-5)
Supplement: Supplementary file 1 — Supplementary Material 1 [file 10096_2025_5109_MOESM1_ESM.docx]

**Title**

Cefiderocol treatment for patients infected by *Stenotrophomonas maltophilia*, *Burkholderia cepacia* complex and *Achromobacter* spp.: subgroup analysis from the PERSEUS study

*Running title: Cefiderocol against rare non-fermenting bacteria*

**Authors**

Julian Torre-Cisneros^1,2,3,4^ · Ricard Ferrer^5^ · Carmen De La Fuente Martos^1,2,6^ · Jessica Sarda^7^ · A. Javier Gonzalez Calvo^7^ · Stefano Verardi^8^ · Andreas Karas^8^ · Alex Soriano^2,9,10^

**Affiliations**

^1^Maimonides Institute for Biomedical Research, Córdoba, Spain;

^2^Centro de Investigación Biomédica en Red de Enfermedades Infecciosas, Instituto de Salud Carlos III, Madrid, Spain;

^3^Infectious Diseases Service, Hospital Universitario Reina Sofía, Córdoba, Spain;

^4^Department of Medical and Surgical Sciences, University of Córdoba, Córdoba, Spain;

^5^Intensive Care Department, Hospital Universitari Vall d'Hebrón, SODIR Research Group, Vall d'Hebron Institut de Recerca, Universitat Autònoma de Barcelona, Barcelona, Spain;

^6^Critical Care Service, Hospital Universitario Reina Sofía, Córdoba, Spain;

^7^Shionogi SLU, Madrid, Spain;

^8^Shionogi BV, London, United Kingdom;

^9^Department of Infectious Diseases, University of Barcelona, Hospital Clinic of Barcelona, Barcelona, Spain;

^10^IDIBAPS, Institut d'Investigacions Biomèdiques Agustí-Pi Sunyer, Barcelona, Spain.

**Corresponding author**

Jessica Sarda

Shionogi S.L.U., Calle de Serrano 45, Madrid, 28001, Spain

Email: jessica.sarda@shionogi.eu

Tel: +34 600 866 774

**Table S1.** Medical history of patients treated with cefiderocol by Gram-negative bacterial species

| Medical history, *n* (%) | *S. maltophilia* | Other non-fermenting Gram-negative bacteria | | |
| --- | --- | --- | --- | --- |
|  | *N*=20 | Total *N*=14^a^ | Bcc *N*=8 | *Achromobacter* spp. *N*=5 |
| Any pre-existing medical condition | 16 (80.0) | 10 (71.4) | 6 (75.0) | 3 (60.0) |
| Tumour (solid organ or haematological) within the last 5 years | 8 (40.0) | 2 (14.3) | 1 (12.5) | 1 (20.0) |
| Diabetes mellitus | 2 (10.0) | 2 (14.3) | 0 (0) | 2 (40.0) |
| Moderate or severe chronic renal disease | 4 (20.0) | 0 (0) | 0 (0) | 0 (0) |
| Chronic obstructive pulmonary disease | 1 (5.0) | 1 (7.1) | 0 (0) | 1 (20.0) |
| Peripheral vascular disease | 1 (5.0) | 2 (14.3) | 1 (12.5) | 1 (20.0) |
| Structural lung disease | 2 (10.0) | 6 (42.9) | 4 (50.0) | 1 (20.0) |
| Congestive heart failure | 2 (10.0) | 0 (0) | 0 (0) | 0 (0) |
| Myocardial infarction | 0 (0) | 1 (7.1) | 1 (12.5) | 0 (0) |
| Chronic liver disease | 2 (10.0) | 1 (7.1) | 1 (12.5) | 0 (0) |
| Cerebrovascular disease | 3 (15.0) | 1 (7.1) | 0 (0) | 1 (20.0) |
| Rheumatic/connective tissue disease | 2 (10.0) | 1 (7.1) | 1 (12.5) | 0 (0) |
| Gastric ulcer | 1 (5.0) | 0 (0) | 0 (0) | 0 (0) |
| Acquired immunodeficiency syndrome | 0 (0) | 1 (7.1) | 0 (0) | 1 (20.0) |
| Hemiplegia or paraplegia | 0 (0) | 0 (0) | 0 (0) | 0 (0) |
| Dementia | 0 (0) | 0 (0) | 0 (0) | 0 (0) |
| Metastatic solid tumour | 0 (0) | 0 (0) | 0 (0) | 0 (0) |

Bcc, *Burkholderia cepacia* complex.
**^a^**One patient had *Ralstonia mannitolilytica* infection (further details not shown), 8 patients had Bcc, 5 patients had *Achromobacter* spp.

**Table S2.** Admission type, hospitalisation and other pathogens

|  | *S. maltophilia* | Other non-fermenting Gram-negative bacteria | | |
| --- | --- | --- | --- | --- |
|  | *N*=20 | Total *N*=14^a^ | Bcc *N*=8 | *Achromobacter* spp. *N*=5 |
| Admission type, *n* (%) |  |  |  |  |
| Emergency | 13 (65.0) | 9 (64.3) | 6 (75.0) | 3 (60.0) |
| Scheduled admission | 6 (30.0) | 4 (28.6) | 1 (12.5) | 2 (40.0) |
| Other | 1 (5.0) | 1 (7.1) | 1 (12.5) | 0 (0) |
| LOS, median (IQR), days | 75.0 (31.0–124.5) | 80.5 (26.0–118.0) | 97.5 (25.0–140.0) | 72.0 (61.0–118.0) |
| ICU LOS, median (IQR), days | 75.0 (18.0–112.0) | 67.0 (24.0–102.0) | 25.0 (16.0–102.0) | 89.0 (72.0–117.0) |
| Creatinine clearance, median (IQR), mL/min | 84.0 (56.0–92.5) | 80.0 (55.0–100.0) | 62.0 (44.5–80.0) | 101.0 (90.0–231.0) |
| Any other pathogen, *n* (%) | 7 (35.0) | 5 (37.5) | 2 (25.0) | 3 (60.0) |
| Gram-positive | 4/7 (57.1) | 2/5 (40.0) | 1/2 (50.0) | 1/3 (33.3) |
| Fungal infection | 4/7 (57.1) | 3/5 (60.0) | 1/2 (50.0) | 2/3 (66.7) |
| Anaerobe infection | 0/7 (0) | 0/5 (0) | 0/2 (0) | 0/3 (0) |

Bcc, *Burkholderia cepacia* complex; ICU, intensive care unit; IQR, interquartile range; LOS, length of stay.
**^a^**One patient had *Ralstonia mannitolilytica* infection (further details not shown), 8 patients had Bcc, 5 patients had *Achromobacter* spp.

**Table S3.** Susceptibility profile of baseline Gram-negative bacterial species^a^

|  | *S. maltophilia* | Other non-fermenting Gram-negative bacteria | | |
| --- | --- | --- | --- | --- |
|  | *N*=20 | Total *N*=14^b^ | Bcc *N*=8 | *Achromobacter* spp. *N*=5 |
| Meropenem susceptibility, *n/N’* (%) | *N*’=8 | *N*’=7 | *N*’=4 | *N*’=2 |
| Resistant | 8 (100) | 7 (100) | 4 (100) | 2 (100) |
| Intermediate | 0 (0) | 0 (0) | 0 (0) | 0 (0) |
| Susceptible | 0 (0) | 0 (0) | 0 (0) | 0 (0) |
| Ceftazidime-avibactam susceptibility, *n/N’* (%) | *N*’=7 | *N*’=5 | *N*’=3 | *N*’=1 |
| Resistant | 7 (100) | 4 (80.0) | 2 (66.7) | 1 (100) |
| Intermediate | 0 (0) | 1 (20.0) | 1 (33.3) | 0 (0) |
| Susceptible | 0 (0) | 0 (0) | 0 (0) | 0 (0) |
| Ceftolozane-tazobactam susceptibility, *n/N’* (%) | *N*’=2 | *N*’=4 | *N*’=2 | *N*’=1 |
| Resistant | 2 (100) | 4 (100) | 2 (100) | 1 (100) |
| Intermediate | N/A | N/A | N/A | N/A |
| Susceptible | 0 (0) | 0 (0) | 0 (0) | 0 (0) |
| Levofloxacin susceptibility, *n/N’* (%) | *N*’=9 | *N*’=1 | N/A | N/A |
| Resistant | 5 (55.6) | 1 (100) | N/A | N/A |
| Intermediate | 0 (0) | 0 (0) | N/A | N/A |
| Susceptible | 4 (44.4) | 0 (0) | N/A | N/A |
| Ciprofloxacin susceptibility, *n/N’* (%) | *N*’=7 | *N*’=10 | *N*’=4 | *N*’=5 |
| Resistant | 7 (100) | 10 (100) | 4 (100) | 5 (100) |
| Intermediate | 0 (0) | 0 (0) | 0 (0) | 0 (0) |
| Susceptible | 0 (0) | 0 (0) | 0 (0) | 0 (0) |
| Minocycline susceptibility, *n/N’* (%) | *N*’=5 | *N*’=3 | *N*’=0 | *N*’=2 |
| Resistant | 0 (0) | 1 (33.3) | 0 (0) | 1 (50.0) |
| Intermediate | 1 (20.0) | 0 (0) | 0 (0) | 0 (0) |
| Susceptible | 4 (80.0) | 2 (66.7) | 0 (0) | 1 (50.0) |
| Trimethoprim-sulfamethoxazole susceptibility, *n/N’* (%)^c,d^ | *N*’=16 | *N*’=8 | *N*’=3 | *N*’=4 |
| Resistant | 6 (37.5) | 5 (62.5) | 1 (33.3) | 4 (100) |
| Intermediate | 4 (25.0) | 1 (12.5) | 1 (33.3) | 0 (0) |
| Susceptible | 6 (37.5) | 2 (25.0) | 1 (33.3) | 0 (0) |
| Ceftazidime susceptibility, *n/N’* (%) | *N*’=14 | *N*’=9 | *N*’=3 | *N*’=5 |
| Resistant | 12 (85.7) | 9 (100) | 3 (100) | 5 (100) |
| Intermediate | 1 (7.1) | 0 (0) | 0 (0) | 0 (0) |
| Susceptible | 1 (7.1) | 0 (0) | 0 (0) | 0 (0) |
| Colistin susceptibility, *n/N’* (%) | *N*’=8 | *N*’=8 | *N*’=3 | *N*’=4 |
| Resistant | 4 (50.0) | 6 (75.0) | 2 (66.7) | 4 (100) |
| Intermediate | N/A | N/A | N/A | N/A |
| Susceptible | 4 (50.0) | 2 (25.0) | 1 (33.3) | 0 (0) |

N’ is the total number of patients with available information.
Bcc, *Burkholderia cepacia* complex; EUCAST, European Committee on Antimicrobial Susceptibility Testing; MIC, minimum inhibitory concentration; N/A, not applicable; TMP-SMX, trimethoprim-sulfamethoxazole.
^a^Information on antibiotic susceptibility was reported by the investigator based on locally obtained microbiological culture and susceptibility testing; no details were collected regarding the nature of the test used or the interpretive criteria.
^b^One patient had *Ralstonia mannitolilytica* infection (further details not shown), 8 patients had Bcc, 5 patients had *Achromobacter* spp.
^c^EUCAST susceptibility interpretive criteria had been updated in January 2020 for TMP-SMX: MICs between >0.001 µg/mL and ≤4 µg/mL or disk zone diameters between <50 mm and ≥16 mm should be interpreted as “susceptible, increased exposure”.
^d^Patients with *S. maltophilia* in this study were treated with cefiderocol between 2021 and 2023.

**Table S4.** Gram-negative antibiotics given prior to cefiderocol treatment

| Prior antibiotics, *n* (%) | *S. maltophilia* | Other non-fermenting Gram-negative bacteria | | |
| --- | --- | --- | --- | --- |
|  | *N*=20 | Total *N*=14^a^ | Bcc *N*=8 | *Achromobacter* spp. *N*=5 |
| Trimethoprim-sulfamethoxazole | 10 (50.0) | 2 (14.3) | 1 (12.5) | 1 (20.0) |
| Meropenem | 6 (30.0) | 4 (28.6) | 3 (37.5) | 1 (20.0) |
| Ceftazidime-avibactam | 5 (25.0) | 3 (21.4) | 3 (37.5) | 0 (0) |
| Colistin | 1 (5.0) | 4 (28.6) | 3 (37.5) | 1 (20.0) |
| Tigecycline | 3 (15.0) | 3 (21.4) | 2 (25.0) | 1 (20.0) |
| Piperacillin-tazobactam | 2 (10.0) | 2 (14.3) | 1 (12.5) | 1 (20.0) |
| Ceftazidime | 1 (5.0) | 2 (14.3) | 2 (25.0) | 0 (0) |
| Aztreonam | 3 (15.0) | 1 (7.1) | 1 (12.5) | 0 (0) |
| Ciprofloxacin | 2 (10.0) | 1 (7.1) | 1 (12.5) | 0 (0) |
| Amikacin | 2 (10.0) | 0 (0) | 0 (0) | 0 (0) |
| Ceftolozane-tazobactam | 0 (0) | 1 (7.1) | 1 (12.5) | 0 (0) |
| Fosfomycin | 0 (0) | 1 (7.1) | 0 (0) | 1 (20.0) |
| Other | 6 (30.0) | 3 (21.4) | 3 (37.5) | 0 (0) |

Bcc, *Burkholderia cepacia* complex.
^a^One patient had *Ralstonia mannitolilytica* infection (further details not shown), 8 patients had Bcc, 5 patients had *Achromobacter* spp.

**Table S5** Concomitant Gram-negative antibiotics during cefiderocol treatment by Gram-negative bacterial species

| Concomitant antibiotics^a^, *n* (%) | *S. maltophilia* | Other non-fermenting Gram-negative bacteria | | |
| --- | --- | --- | --- | --- |
|  | *N*=20 | Total *N*=14^b^ | Bcc *N*=8 | *Achromobacter* spp. *N*=5 |
| Trimethoprim-sulfamethoxazole | 5 (25.0) | 2 (14.3) | 1 (12.5) | 1 (20.0) |
| Colistin | 0 (0) | 4 (28.6) | 3 (37.5) | 1 (20.0) |
| Tigecycline | 2 (10.0) | 3 (21.4) | 2 (25.0) | 1 (20.0) |
| Meropenem | 3 (15.0) | 2 (14.3) | 2 (25.0) | 0 (0) |
| Ceftazidime-avibactam | 0 (0) | 3 (21.4) | 3 (37.5) | 0 (0) |
| Ceftazidime | 0 (0) | 2 (14.3) | 2 (25.0) | 0 (0) |
| Piperacillin-tazobactam | 1 (5.0) | 1 (7.1) | 1 (12.5) | 0 (0) |
| Ciprofloxacin | 1 (5.0) | 1 (7.1) | 1 (12.5) | 0 (0) |
| Aztreonam | 0 (0) | 1 (7.1) | 1 (12.5) | 0 (0) |
| Ceftolozane-tazobactam | 0 (0) | 1 (7.1) | 1 (12.5) | 0 (0) |
| Amikacin | 1 (5.0) | 0 (0) | 0 (0) | 0 (0) |
| Imipenem | 0 (0) | 0 (0) | 0 (0) | 0 (0) |
| Fosfomycin | 0 (0) | 0 (0) | 0 (0) | 0 (0) |
| Other | 3 (15.0) | 3 (21.4) | 3 (37.5) | 0 (0) |

Bcc, *Burkholderia cepacia* complex.
^a^Concomitant antibiotics include agents with activity against Gram-negative bacteria, which were initiated prior to cefiderocol and continued during cefiderocol treatment or were initiated simultaneously or were administered following initiation of cefiderocol treatment.
^b^One patient had *Ralstonia mannitolilytica* infection (further details not shown), 8 patients had Bcc, 5 patients had *Achromobacter* spp.
